# Sudden sensorineural hearing loss and vertigo associated with arterial occlusive disease: three case reports and literature review

**DOI:** 10.1590/S1516-31802007000300012

**Published:** 2007-05-03

**Authors:** Ney Penteado de Castro, Clemente Isnard Ribeiro de Almeida, Carlos Alberto Herrerias de Campos

**Keywords:** Deafness, Vertigo, Carotid artery diseases, Ischemia, Dizziness, Surdez, Vertigem, Doenças das artérias carótidas, Isquemia, Tontura

## Abstract

Sudden sensorineural hearing loss and vertigo (SSNHLV) has multifactorial causes, of which viral, autoimmune and vascular insufficiency are the most common. The therapeutic management for SSNHLV includes antiviral drugs, corticosteroids, vasodilators, normovolemic hemodilution therapy and hyperbaric oxygen therapy. Vertebrobasilar occlusive disease and carotid occlusive disease are seldom related to SSNHLV. Discussions concerning SSNHLV caused by occlusive vascular disease are important and necessary for both neurologists and otolaryngologists, since their therapeutic management and prognosis are very different from other causes of hearing loss and vertigo. Here, we present our experience with three cases managed with interventional treatment and conduct a review and discussion on the relevant literature. We conclude that investigation of vertebrobasilar and carotid occlusive diseases is necessary in patients over 50 years of age who present SSNHLV, mild neurological symptoms and a history of arteriosclerosis, high blood pressure or thrombosis.

## RESUMO

Surdez sensorioneural súbita e vertigem súbita (SSNVS) podem ter múltiplas causas, e a infecção virótica, a doença autoimune e a insuficiência vascular são as mais comuns. O tratamento da SSNVS inclui drogas antiviróticas, corticosteróides, vasodilatores, terapia de hemodiluição normovolêmica e terapia hiperbárica. Raramente são relacionadas como causa a doença vertebrobasilar oclusiva e a doença carotídea oclusiva. Discutir SSNVS é importante e necessário para neurologistas e otolaringologistas, uma vez que a terapia e o prognóstico são muito diferentes conforme a etiologia. Apresentamos nossa experiência com três casos em que foi administrado tratamento intervencional para SSNVS de causa oclusiva em grandes vasos e apresentamos uma revisão e discussão da literatura pertinente. Concluímos que a investigação de doenças oclusivas vertebrobasilar e carotídea é necessária em pacientes acima dos 50 anos de idade com SSNVS, sintomas neurológicos moderados e uma história de arteriosclerose, pressão alta ou trombose.

## INTRODUCTION

Sudden sensorineural hearing loss and vertigo (SSNHLV) is defined as a hearing deficiency of sudden onset that develops rapidly over a maximum of 72 hours. The intensity of the hearing deficiency can vary from mild (a minimum of 30 dB of hearing deficiency at three adjacent frequencies) to severe (more than 80 dB), without any history of hearing fluctuation and with predominantly sensorial characteristics. It may be associated with vestibular symptoms. Sometimes only a vestibular syndrome appears. The cause may be multifactorial, usually affecting the microcirculation of the inner ear and consequently impairing cochleovestibular function. The most frequent causes are viral infection and autoimmune disease; however, neurilemmoma of the VIII nerve and the labyrinthine fistulae should also be considered. The therapeutic approach involves the use of corticosteroids, vasodilators, normovolemic hemodilution, hyperbaric oxygen therapy and antiviral drugs.^1^

The worldwide increase in life expectancy makes cerebrovascular diseases more prevalent and may induce other medical conditions such as SSNHLV. Occlusive diseases of the vertebrobasilar system and carotid artery obstructive disease may reduce the blood flow through the labyrinth artery, thus leading to cochleovestibular manifestations such as sudden hearing loss and vertigo, which may be associated with symptoms relating to ischemia of the cerebral trunk.^2–9^

## OBJECTIVE

The objective of this paper was to present three cases of SSNHLV related to carotid and vertebrobasilar obstructive disease and discuss the literature.

## METHODS

We present three cases of SSNHLV followed by the authors and present a literature review on the subject. The earliest reported case of SSNHLV with nuclear magnetic resonance (NMR) angiography imaging is from 1993. Therefore, a retrospective review of the literature between the years 1993 and 2005 was conducted using key words such as “sudden sensorineural hearing loss”, “vertebral artery”, “carotid artery” and “human” was conducted on the Medical Literature Analysis and Retrieval System Online (Medline), Literatura Latino-Americana e do Caribe em Ciências da Saúde (Lilacs), Scientific electronic library online (SciELO) and Cochrane databases We found 423 references in Medline, eight references in Lilacs, three references in SciELO and two references in Cochrane. The parameters examined were incidence, clinical findings, vascular findings, diagnosis and treatment.

### Case presentation

**Figure 1:**
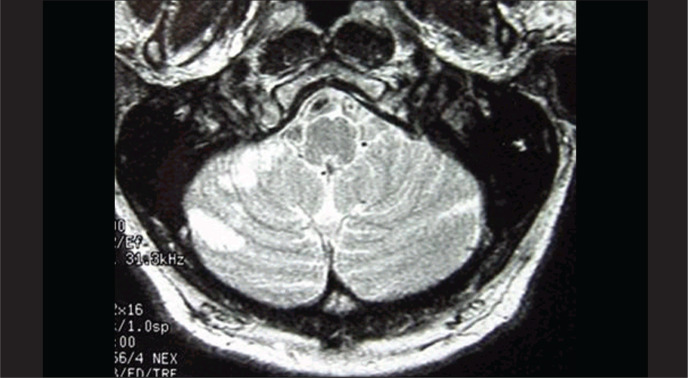
Case 1: encephalic nuclear magnetic resonance (NMR) showing signs of senile angiopathy and ischemic areas on the right cerebellar hemisphere.

**Figure 2:**
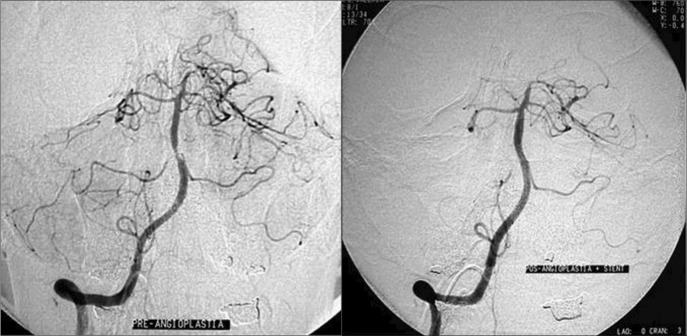
Case 1: right vertebral artery and basilar trunk arteriography. Before (left) and after (right) angioplasty and stenting of the vertebral and basilar arteries.

*Case 1*: This was a 68-year-old businessman who complained of sudden intense dizziness accompanied by significant disabling imbalance, nausea and profuse sudoresis, associated with a crisis of systemic arterial hypertension. He reported having suffered mild hearing loss in the right ear after a cerebral ischemic accident three years earlier. His personal history included systemic arterial hypertension, diabetes mellitus type II and dyslipidemia. The evaluation on the first day revealed the presence of mild sensorineural hearing loss in the right ear, spontaneous directional nystagmus with horizontal rotation to the left, and horizontal deviation to the right in the index-to-index test. Although the arterial hypertension and diabetes mellitus were under control and despite administering cinnarizine 150 mg/day and clonazepam 1 mg/day, the dizziness was only slightly better on the third day. At this point, the patient presented sudden hearing loss and intense tinnitus in the right ear, with worsening of the dizziness. The neurological evaluation on this day showed mild cerebellar ataxia and encephalic nuclear magnetic resonance (NMR) revealed recent areas of ischemic lesion in the right cerebellar hemisphere (Figure 1). After introducing anticoagulants for one week the patient showed mild improvement in the vestibular symptoms, but the hearing and tinnitus in the right ear and the cerebellar ataxia remained unaffected. NMR angiography showed decreased blood flow at the origin of the vertebral arteries and in the trunk of the basilar artery. Angioplasty was accomplished with stenting at the origin of the right vertebral artery and in the middle third of the basilar artery (Figure 2). The patient presented significant improvement in his vertigo, tinnitus and hearing four days after the procedure.

**Figure 3:**
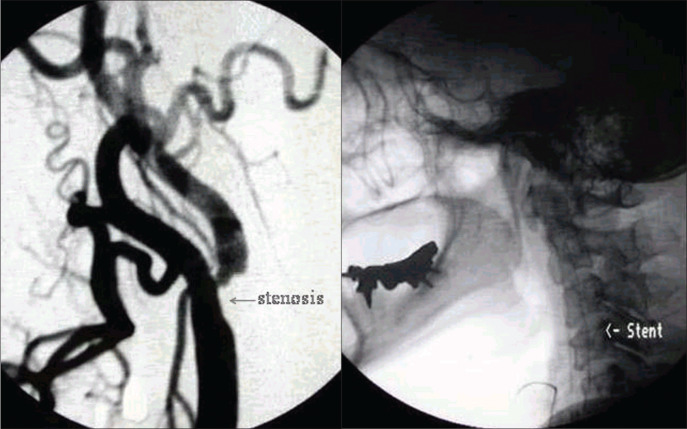
Case 2: Right carotid arteriography showing significant bulb stenosis before angioplasty (left) and its correction after angioplasty (right).

*Case 2*: This was a 62-year-old woman who, two days after a hypertensive crisis (220/170 mmHg) that was controlled with beta-blocker, presented mild to moderate dizziness when lying down, in spite of the use of cinnarizine, clonazepam and gingko biloba (EGB 761). One month later, she presented sudden moderate hearing loss with tinnitus in the right ear. Neurological evaluation revealed severe vestibular deficit (responding only to 30° C in the right ear. Vertebrobasilar echo-Doppler showed significant obstruction due to atheromatosis in the right carotid bulb. NMR angiography showed significant stenosis of the right bulb and moderate stenosis in the intradural portion of the vertebral arteries and basilar trunk. Selective arteriography of the right carotid confirmed the stenosis close to the bulb, and this was corrected by angioplasty with stenting (Figure 3).

Following this procedure, the patient presented significant improvement in the hearing loss and dizziness, with persistence of the tinnitus but at lower intensity. Otoneurological evaluation one month later showed that the hearing parameters were normal, while the vestibular deficit in the right ear persisted.

*Case 3*: This was a 78-year-old man with diabetes mellitus type II and compensated cardiac supraventricular arrhythmia who had experienced frequent syncopes four years earlier. He presented severe vestibular syndrome for four days and was admitted to hospital for treatment. The otoneurological evaluation revealed right vestibular areflexia in the Kobrack test and normal acumetry. A hypothesis of sudden vertigo of probable vascular origin was established because of the patient's history. NMR angiography showed no flow in the right vertebral artery, starting from its origin in the right subclavian artery. Arteriography confirmed the lack of vascular patency, and this was corrected by placement of an arterial stent. On the third day after the procedure, the patient presented significant improvement in his clinical picture and in his vestibular symptoms.

## DISCUSSION

There are only ten studies in the literature referring to an association between SSNHLV and vertebrobasilar obstructive disease (Table 1) and three papers referring to an association with carotid obstructive disease (Table 2).

**Table 1.** Papers reporting cases of occlusive vertebrobasilar disease in association with sudden sensorineural hearing loss

Year	Authors	Study design	VBAOD N/total	Comments
1993	Yamasoba et al.^2^	Prospective	12/57	NMR: low vertebrobasilar flow Sensorineural hearing loss and electronystagmography abnormal Headache and hypoesthesia of the ear Age: patients over 50 years old
1994	Kido et al.^6^	Case report	1	NMR: anteroinferior cerebellar artery and posteroinferior cerebellar artery ischemia Sensorineural hearing loss and vestibular areflexia Total spontaneous recovery at 14^th^ day
1995	Schweizer et al.^14^	Prospective	98	Vertebral echo-Doppler Normal examination
1999	Otterstedde et al.^12^	Case report	1	NMR angiography: megadolichobasilar vascular compression of the VIII nerve Age: 71 years old
2000	Schmiz et al.^5^	Case report	4	NMR angiography: basilar thrombosis Sensorineural hearing loss and vertigo
2000	De Felice et al.^15^	Prospective	12/22	Transcranial echo-Doppler: comparison between 22 patients with sensorineural hearing loss and 41 controls Non-functioning posterior communicating artery in the Willis polygon 12 patients with sensorineural hearing loss versus 4 controls
2002	Lee et al.^4^	Prospective	12	NMR angiography: ischemia of anteroinferior cerebellar artery, sensorineural hearing loss and vestibular areflexia (83%)
2003	Dziewas et al.^13^	Case report	1	NMR: paramedian pontine ischemia Megadolichobasilar thrombosis
2004	Sauvaget et al.^3^	Series	4/333	NMR: VBAOD Sensorineural hearing loss and late neurological symptoms

*VBAOD = vertebrobasilar artery occlusive disease; NMR = nuclear magnetic resonance.*

**Table 2.** Papers reporting cases of occlusive carotid disease in association with sudden sensorineural hearing loss

Year	Authors	Study design	CAOD N/total	Comments
1993	Perie et al.^7^	Case report	2/3	Sensorineural hearing loss Pulsatile pharyngeal lateral wall Vascular risk factors NMR and echo-Doppler: Carotid “megadolicho”
1997	Ohinata et al.^8^	Randomized clinical trial	14/70	Vertebral and carotid echo-Doppler 14 patients with sensorineural hearing loss versus 70 controls Carotid and vertebral blood flow lower in patients with more than 50 dB loss
1998	Steidtmann et al.^9^	Case report	1	Internal carotid artery dissection

*CAOD = carotid artery occlusive disease; NMR = nuclear magnetic resonance.*

### Incidence

The incidence of SSNHLV is between 5 and 25 cases per 100,000 inhabitants per year^1,10,11^ and of these cases, 1.2%-21% are caused by vertebrobasilar obstructive disease.^2,3^ This disease shows greater prevalence among individuals over 50 years of age. Among such individuals it has been attributed to vertebrobasilar artery insufficiency,^2^ arteriosclerosis and thromboembolic events,^3,5^ acute ischemia of the anteroinferior cerebellar artery^4,6^ or megadolichobasilar artery.^12,13^

### Clinical findings

Cochleovestibular symptoms usually precede neurological symptoms. Vertigo and sudden deafness are the most common manifestations. The hearing loss is bilateral in most cases, with sensorineural characteristics.^5,6^ Electronystagmography reveals vestibular areflexia in 83% of the cases.^4,5^ Cochleovestibular symptoms are attributed to low flow in the labyrinth artery. Occipital headache, diplopia, cerebellar ataxia, facial hemiparesis and hypoesthesia, dysphasia and dysphonia may be present.^2,3,12^

### Vascular findings

An association between lack of carotid artery patency and SSNHLV is rare. These are “megadolicho” conditions of the carotid artery,^7^ overall cerebrovascular insufficiency^8^ and carotid artery dissection.^9^

### Diagnosis

The most useful tests for vertebrobasilar obstructive disease are NMR, NMR angiography and vertebrobasilar arteriography. In a prospective study on 98 patients with SSNHLV, vertebral echo-Doppler did not demonstrate any changes.^14^ In another study, comprising 22 patients with sensorineural hearing loss, transcranial echo-Doppler detected 55% (12/22) of the cases of low flow in the posterior communicating homolateral artery of the Willis polygon.^15^ However, a comparative study using transcranial echo-Doppler on 27 normal subjects and 27 patients with SSNHLV did not show any correlation between hearing status and low flow in the posterior communicating artery. This test was not recommended for evaluating the cause of SSNHLV.^16^ The preferred tests for identifying ischemic areas in the cerebellar-pontine region are NMR and NMR angiography.^2–13,17^

### Etiology and treatment

The basic clinical therapy consists of anticoagulants and vasodilators to reestablish arterial flow and minimize the harmful effect of ischemia.^2–6,15^ SSNHLV is a disease of multifactorial etiology, with vertebrobasilar obstructive disease as one of the main causes. The variability in the prevalence of this condition (1.2 to 21%) is probably due to the different age groups studied; an association between SSNHLV and vertebrobasilar obstructive disease is expected to be more likely among the elderly. All of the five prospective studies that have been conducted had SSNHLV as a sample inclusion criteria. The patients in these studies underwent vertebrocarotid echo-Doppler or encephalic NMR angiography to detect vertebrobasilar or carotid diseases that would be related to cochleovestibular symptoms.

Cochleovestibular symptoms occur mainly due to ischemia of the labyrinth artery. Dysacusia and vestibular symptoms are among the most common inner ear symptoms reported,^2–5^ although they may occur in association with ischemic images on NMR in the cerebellar-pontine areas, which may produce alterations of central character. In most studies, the clinical description was of sudden hearing loss and severe vertigo, thus demonstrating vestibular areflexia in 83% of the cases.^4^ Occipital headache, diplopia, cerebellar ataxia, hemiparesis, facial hypoesthesia, dysphasia and dysphonia may be associated symptoms.^2,3,18^

NMR and NMR angiography are the most useful techniques for demonstrating arterial diseases of the vertebrobasilar trunk.^2–6,9,13,17^ Although vertebrobasilar arteriography is an invasive procedure, it should be used to confirm vertebrobasilar obstructive disease, thereby avoiding false positive cases detected by NMR and NMR angiography.^1,2^

Carotid artery occlusion that induces SSNHLV is rare, but is possible especially when associated with vertebrobasilar inadequacy.^7–9^ On the other hand, obstructive vertebrobasilar artery disease with significantly reduced carotid flow (decrease of more than 60%, on echo-Doppler) may impair the microcirculation of the internal auditory artery, thus causing SSNHLV.

SSNHLV as a result of occlusive cerebrovascular disease should be suspected when: 1) the patient is over 50 years of age; 2) there is vertigo preceding the crisis; 3) there are metabolic risk factors that induce arteriosclerosis; or 4) there is a history of arteriosclerosis, systemic arterial hypertension or cerebrovascular accident.

## CONCLUSION

When a diagnosis of occlusive cerebrovascular disease is established, discussions with the hemodynamic team are necessary in order to evaluate the benefits and risks of interventional therapy. Such therapy, when performed correctly, can improve the quality of life for these patients, and therefore represents an excellent therapeutic option.
